# Therapeutic potential and functional interaction of carfilzomib and vorinostat in T-cell leukemia/lymphoma

**DOI:** 10.18632/oncotarget.8667

**Published:** 2016-04-09

**Authors:** Minjie Gao, Gege Chen, Houcai Wang, Bingqian Xie, Liangning Hu, Yuanyuan Kong, Guang Yang, Yi Tao, Ying Han, Xiaosong Wu, Yiwen Zhang, Bojie Dai, Jumei Shi

**Affiliations:** ^1^ Department of Hematology, Shanghai Tenth People's Hospital, Tongji University School of Medicine, Shanghai, China; ^2^ College of Life Science and Technology, Tongji University, Shanghai, China

**Keywords:** carfilzomib, vorinostat, T-cell leukemia and lymphoma

## Abstract

We previously showed that the proteasome inhibitor carfilzomib and the histone deacetylase inhibitor (HDACI) vorinostat cooperated to induce cell apoptosis in one T-cell leukemia cell line *in vitro*, implying the possibility of the combination treatment *of* carfilzomib and vorinostat as a potential therapeutic strategy in human T-cell leukemia/lymphoma. Here we report that combination treatment of carfilzomib and vorinostat enhanced cell apoptosis and induced a marked increase in G_2_-M arrest, reactive oxygen species (ROS) generation, and activated the members of mitogen-activated protein kinases (MAPK) family, including the stress-activated kinases JNK, p38MAPK, and ERK1/2. Carfilzomib/vorinostat-mediated apoptosis was blocked by the ROS scavenger N-acetylcysteine (NAC). The JNK inhibitor SP600125 and the p38MAPK inhibitor SB203580 but not the MEK1/2 inhibitor U0126 significantly attenuated carfilzomib/vorinostat-induced apoptosis, suggesting that p38MAPK and JNK activation contribute to carfilzomib and vorinostat-induced apoptosis. This was further confirmed via short hairpin (shRNA) RNA knockdown of p38MAPK and JNK. Interestingly, the ROS scavenger NAC attenuated carfilzomib/vorinostat-mediated activation of p38MAPK and JNK. However, p38MAPK shRNA but not JNK shRNA diminished carfilzomib/vorinostat-mediated ROS generation. In contrast, overexpression of p38MAPK significantly increased carfilzomib/vorinostat-mediated ROS generation, suggesting that an amplification loop exists between ROS and p38MAPK pathway. Combination treatment of carfilzomib and vorinostat enhanced their individual antitumor activity in both a human xenograft model as well as human primary T-cell leukemia/lymphoma cells. These data suggest the potential clinical benefit and underlying molecular mechanism of combining carfilzomib with vorinostat in the treatment of human T-cell leukemia/lymphoma.

## INTRODUCTION

T-cell acute lymphoblastic leukemia and T-cell lymphoma are aggressive hematopoietic tumors. With the development of intensified chemotherapy, the prognosis of T-cell leukemia/lymphoma has gradually improved. However, the outcome of these patients with relapse and resistance remains extremely poor [1, 2]. Therefore, further research is required to identify therapeutic targets to develop more effective and less toxic antitumor drugs. Multiple signaling pathways are involved in the pathogenesis and survival of T-cell malignancies, such as the dysregulation of NOTCH1 [3, 4], extracellular signal-regulating kinase1/2 (ERK1/2) [5], phosphoinositide 3-kinase (PI3K)-AKT [6], c-jun N-terminal kinase (JNK) [7], p38 mitogen-activated protein kinase (p38MAPK) [7, 8], and Janus kinase/ signal transducers and activators of transcription (JAK/STAT) [9, 10]. Yet much remains to be understood about the role of these signaling pathways in T-cell leukemia/lymphoma.

Proteasome inhibitors are currently used as an effective approach to kill cancer cells which are resistant to conventional chemotherapy. Bortezomib is a first-in-class proteasome inhibitor used either alone or in combination with other agents in the treatment of multiple myeloma (MM) [11]. The preexistence or development of bortezomib resistance, however, has prompted the development of the second-generation proteasome inhibitor carfilzomib, which shows efficacy as either single agent or in combination with other agents against MM [12] and other cancers [13]. Carfilzomib has overcome bortezomib resistance in patients [12] and has been approved for treatment of MM [14].

Another class of drugs, histone deacetylase inhibitors (HDACIs), has also been shown to inhibit cell proliferation, induce differentiation and cell cycle arrest, and promote apoptosis in a wide range of hematological and solid malignancies. These inhibitors have been shown to inhibit cancer cell growth through both the inhibition of histones deacetylation and effects on non-histone proteins [15]. HDACIs alter expression in 2–10% of genes involved in biological processes [16]. Due to the multiple anticancer mechanisms of HDACIs, there is growing interest in exploring permutations of combined therapies in an attempt to maximize the antitumor effect with many of these having been evaluated in pre-clinical *in vivo* models and clinical trials [15]. HDACIs are well-tolerated in a variety of malignancies [15] and vorinostat is an HDACI that has been approved for the treatment of cutaneous T-cell lymphomas [17] making it an attractive candidate.

Synergy between the proteasome inhibitor bortezomib and HDACIs has been described in diverse malignant cell types [18–20], particularly those of hematopoietic origin [21–23], as well as in a number of cancers such as nasopharyngeal carcinoma [18], prostate cancer [24], glioblastoma [25], ovarian carcinoma [26], multiple myeloma [27], acute myeloid leukemia, myelodysplastic syndrome [23], and others. However, bortezomib use can be limited because of peripheral neuropathy and the existence and development of resistance [28]. Carfilzomib, a second-generation, irreversible, selective proteasome inhibitor, was found to be more potent than bortezomib in both MM cell line models and clinical samples [29, 30]. Importantly, carfilzomib had activity against bortezomib-resistant cell lines and bortezomib-resistant primary cells [28, 31]. Thus, the combination of carfilzomib with HDACIs, such as vorinostat, holds promise to be more efficacious and safer than the combination of bortezomib and HDACIs. This combination currently has only been reported in diffuse large-B-cell lymphoma and mantle cell lymphoma [32–33]. However, it has not been well investigated in T-cell leukemia/lymphoma. Our lab has previously observed that the combination has potentiated the apoptosis in Jurkat cell line [34]. Here, we further determined whether combined treatment of carfilzomib and vorinostat has enhanced antitumor activity in *in vitro* other T-cell leukemia/lymphoma cell lines and *in vivo*, and then identified the underlying molecular mechanism. Our results indicated that concurrent administration of carfilzomib and vorinostat enhanced their individual antitumor activity in both a human xenograft model as well as primary human T-cell leukemia/lymphoma cells, in association with the amplification loop between ROS generation and p38MAPK and with ROS-dependent JNK activation. Furthermore, combined treatment of carfilzomib and vorinostat is very well tolerated and only shows minimal toxicity. Collectively, these findings provide a rationale for using this combinatorial therapy in human T-cell leukemia/lymphoma.

## RESULTS

### Carfilzomib and vorinostat effectively inhibited cell proliferation in T-cell leukemia/lymphoma cell lines

**Figure 1:**
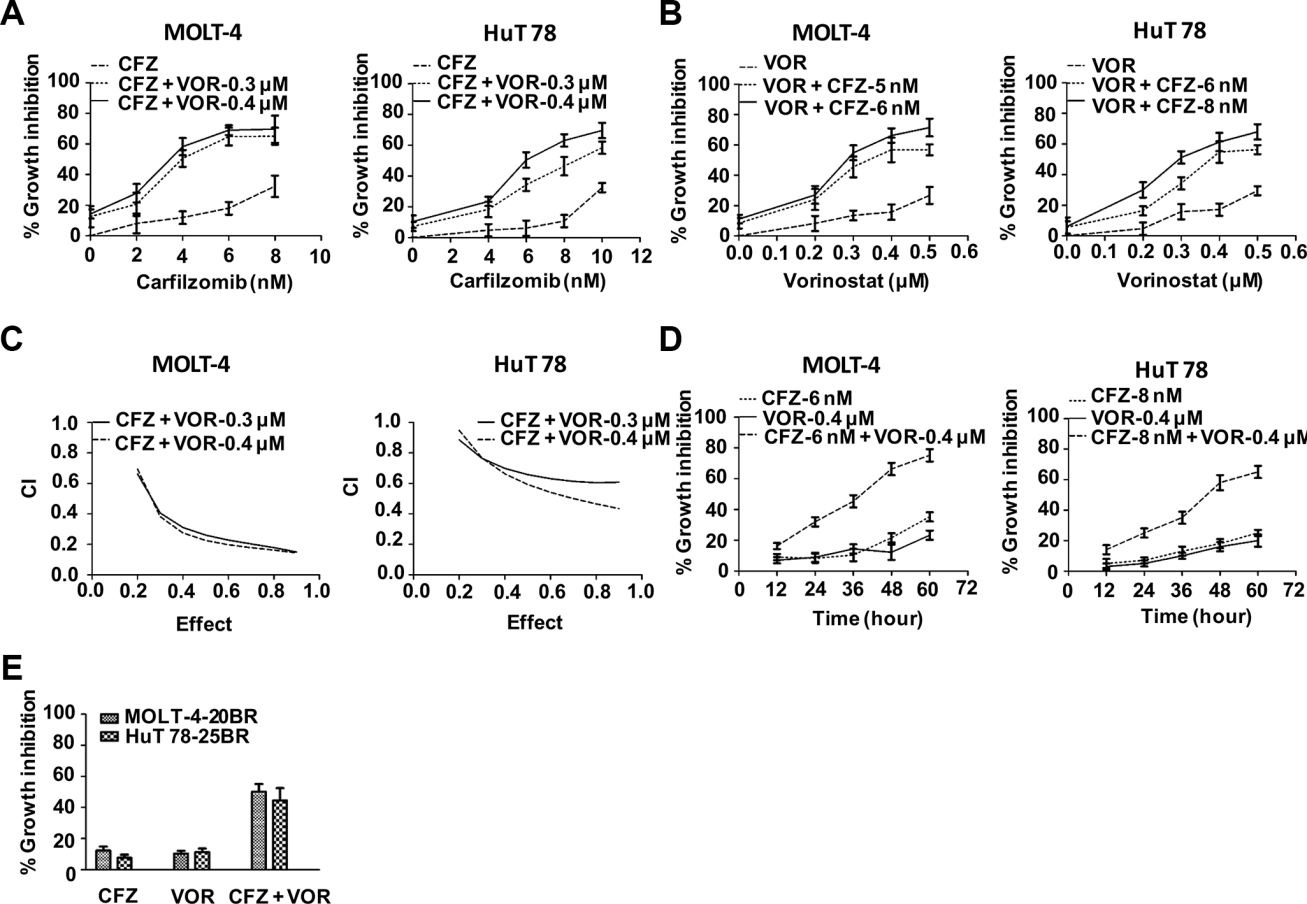
Carfilzomib and vorinostat cooperatively inhibited cell proliferation in T-cell leukemia/lymphoma cell lines (**A**) Cells were treated with various carfilzomib concentrations (MOLT-4 2.0–8.0 nM, HuT 78 4.0–10.0 nM) and fixed vorinostat concentrations (0.3 or 0.4 μM) for 48 h, then cell viability was monitored by CCK-8. (**B**) Cells were treated with various vorinostat concentrations (0.2–0.5 μM) and fixed carfilzomib concentrations (MOLT-4 5 or 6 nM, HuT 78 6 or 8 nM) for 48 h, then cell viability was monitored by CCK-8. (**C**) CI values were calculated based on median-effect principle. CI values less than 1.0 denote synergistic interactions. Results are the means of three experiments. (**D**) Cells were treated with carfilzomib (MOLT-4 6 nM, HuT 78 8 nM) or/and vorinostat (MOLT-4 0.4 μM, HuT 78 0.4 μM) for the indicated intervals, then cell viability was monitored by CCK-8. (**E**) Cells were treated with carfilzomib (MOLT-4-20BR 8 nM, HuT 78-25BR 10 nM) or/and vorinostat (MOLT-4-20BR 0.5 μM, HuT 78-25BR 0.5 μM) for 48 h, then cell viability was monitored by CCK-8. CFZ, carfilzomib; VOR, vorinostat.

To assess the combined effect of carfilzomib and vorinostat, we first evaluated proliferation by CCK-8 assay in the T-cell leukemia and lymphoma cell lines MOLT-4 and HuT 78, respectively, in the presence of either drug alone or their combination. At the concentration of less than 8.0 nM, carfilzomib only modestly inhibited proliferation in both cell lines. In contrast, cell proliferation was substantially inhibited when carfilzomib was combined with low amounts of vorinostat (0.3 or 0.4 μM) (Figure 1A). Similarly, vorinostat on its own was only marginally effective at inhibiting proliferation in both MOLT-4 and HuT78 cells, but exhibited marked inhibition of proliferation in combination with low concentrations of carfilzomib (5 or 6 nM) (Figure 1B). Median dose effect analysis of the interaction between carfilzomib (2–10 nM) and vorinostat (0.3 or 0.4 μM) yielded combination index (CI) values substantially less than 1.0, denoting synergy (Figure 1C). To explore the combined effect over time, both cell lines were treated for various intervals with each drug alone or the combination. This resulted in the most pronounced synergistic effect at 48 h (Figure 1D). In order to test whether combined treatment of carfilzomib and vorinostat were also effective in cell lines which are resistant to bortezomib, MOLT-4-20BR and HuT 78- 25BR bortezomib-resistant cells were generated. Bortezomib-resistance did not abrogate the effect of carfilzomib and vorinostat, but did necessitate slightly higher concentrations (8 nM with 0.5 μM, 10 nM with 0.5 μM, carfilzomib and vorinostat, respectively, at 48 h) (Figure 1E). Together, these data demonstrate that the combination of carfilzomib and vorinostat is more effective against these T-cell leukemia/lymphoma cell lines compared with either inhibitor alone.

### Combination treatment of carfilzomib and vorinostat induces more apoptosis in T-cell leukemia/lymphoma cell lines

**Figure 2:**
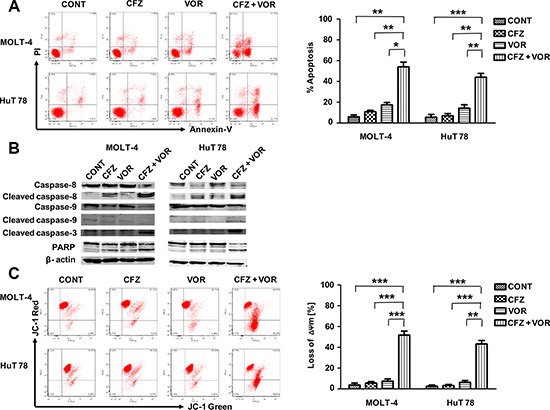
Combination treatment of carfilzomib and vorinostat enhanced apoptosis in T-cell leukemia/lymphoma cell lines (**A**) Cells were treated with carfilzomib (MOLT-4 6 nM, HuT 78 8 nM) or/and vorinostat (MOLT-4 0.4 μM, HuT 78 0.4 μM) for 48 h, then cell apoptosis was monitored by Annexin V/PI staining. Columns represent the average percent of Annexin V positive cells from three independent experiments, which are shown as the mean ± SD. (**B**) After treatment as in A, the expression of caspase-8, cleaved caspase-8, caspase-9, cleaved caspase-9, cleaved caspase-3 and PARP were monitored by western blot. (**C**) After 24 h of drug exposure as in panel A, stained with JC-1 dye, mitochondrial membrane potential was detected by flow cytometry. Only JC-1 green positive (lower right quadrant) cells were analyzed for the loss of mitochondrial membrane potential. Columns represent the average percent of only JC-1 green positive cells from three independent experiments, which are shown as the mean ± SD. CFZ, carfilzomib; VOR, vorinostat; ΔΨm, mitochondrial membrane potential. *represent *p* < 0.05; **represent *p* < 0.01; ***represent *p* < 0.001.

To explore whether the combination of carfilzomib and vorinostat induced more apoptosis, MOLT-4 and HuT 78 cells were incubated with carfilzomib (MOLT-4 6 nM, HuT 78 8 nM) or/and vorinostat (MOLT-4 0.4 μM, HuT 78 0.4 μM) for 48 h and cell apoptosis was analyzed by flow cytometry using Annexin V staining kit. Combination treatment resulted in a significant percentage of apoptotic cells, 59.1% ± 4.6% in MOLT-4 cells and 50.9% ± 3.5% in HuT 78 cells respectively, whereas the percentage of apoptotic cells upon treatment with carfilzomib alone (MOLT-4 11.2% ± 1.8%, HuT 78 11.0% ± 1.9%) or vorinostat alone (MOLT-4 13.5% ± 1.8%, HuT 78 15.7% ± 2.4%) was considerably lower (Figure 2A). Cell apoptosis was further confirmed by caspase activation and PARP cleavage (Figure 2B). Using the fluorescent probe JC-1, a sensitive marker for mitochondrial membrane potential, combination treatment substantially reduced mitochondrial membrane potential in MOLT-4 and HuT 78 cells compared to either carfilzomib or vorinostat alone (Figure 2C). Together these findings demonstrate that combination treatment of carfilzomib and vorinostat induces more apoptosis than either treatment alone.

### MAPK signaling pathways were modulated by combination treatment of carfilzomib and vorinostat

**Figure 3:**
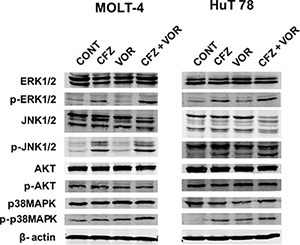
MAPK signaling pathways are mediated by combination treatment of carfilzomib and vorinostat Cells were treated with carfilzomib (MOLT-4 6 nM, HuT 78 8 nM) or/and vorinostat (MOLT-4 0.4 μM, HuT 78 0.4 μM) for 48 h, then the expression of phospho (p)-AKT, AKT, p-JNK, JNK, p-ERK1/2, ERK1/2, p-p38MAPK and p38MAPK were monitored by western blot. CFZ, carfilzomib; VOR, vorinostat.

Multiple signaling pathways were shown to be involved in the apoptosis of cancer cells induced by the combination of proteasomal and histone deacetylase inhibition, including the MAPK family [33, 35, 36] and AKT [37]. As shown in Figure 3, treatment with carfilzomib alone resulted in activation of p38MAPK, JNK and ERK1/2 and the combination treatment potentiated the activation in both cell types. The combination treatment didn't change the phosphorylation of AKT (Figure 3). These data suggest that vorinostat potentiates the activation of p38MAPK, JNK and ERK1/2 by carfilzomib.

### Carfilzomib in combination with vorinostat potentiates G_2_-M arrest

To study the effect of carfilzomib and vorinostat on the cell cycle, cells were incubated with carfilzomib (MOLT-4 6 nM, HuT 78 8 nM) and/or vorinostat (MOLT-4 0.4 μM, HuT 78 0.4 μM) for 24 h, and cell cycle was analysed by flow cytometry. As shown in Table 1, cells treated with vorinostat alone slightly increased the S phase population in both cell lines. Cells treated with carfilzomib alone accumulated in G_2_-M phase of the cell cycle in both cell lines. While cells were treated with the combination of carfilzomib with vorinostat, there was a highly significant increase in the G_2_-M phase population, compared to treatment with carfilzomib alone (*n* = 3, *P* < 0.005).

**Table 1:** Combination of carfilzomib with vorinostat induced G_2_-M arrest

Cell cycle	Control	CFZ	VOR	CFZ and VOR
MOLT-4				
G_0_G_1_	44.9 + 3.1	22.8 + 1.7	44.8 + 2.7	35.8 + 3.1
G_2_-M	12.3 + 1.5	20.2 + 2.1**	6.8 + 1.7	35.3 + 3.6^†^
S	42.7 + 2.6	57.1 + 3.1	48.3 + 2.3	29.0 + 4.9
HuT 78				
G_0_G_1_	54.3 + 2.7	44.2 + 1.7	54.0 + 2.7	41.2 + 1.6
G_2_-M	10.1 + 1.4	16.7 + 1.3**	6.3 + 1.2	38.0 + 2.6^†^
S	35.6 + 3.7	39.2 + 0.7	39.7 + 3.6	20.8 + 1.0

** represent significant differences relative to controls (*p* < 0.01);

† represent significant differences relative to CFZ (*p* < 0.001).

CFZ, carfilzomib; VOR, vorinostat.

### Combination treatment induced ROS generation and the increase of ROS generation played a critical role in the induction of apoptosis

**Figure 4:**
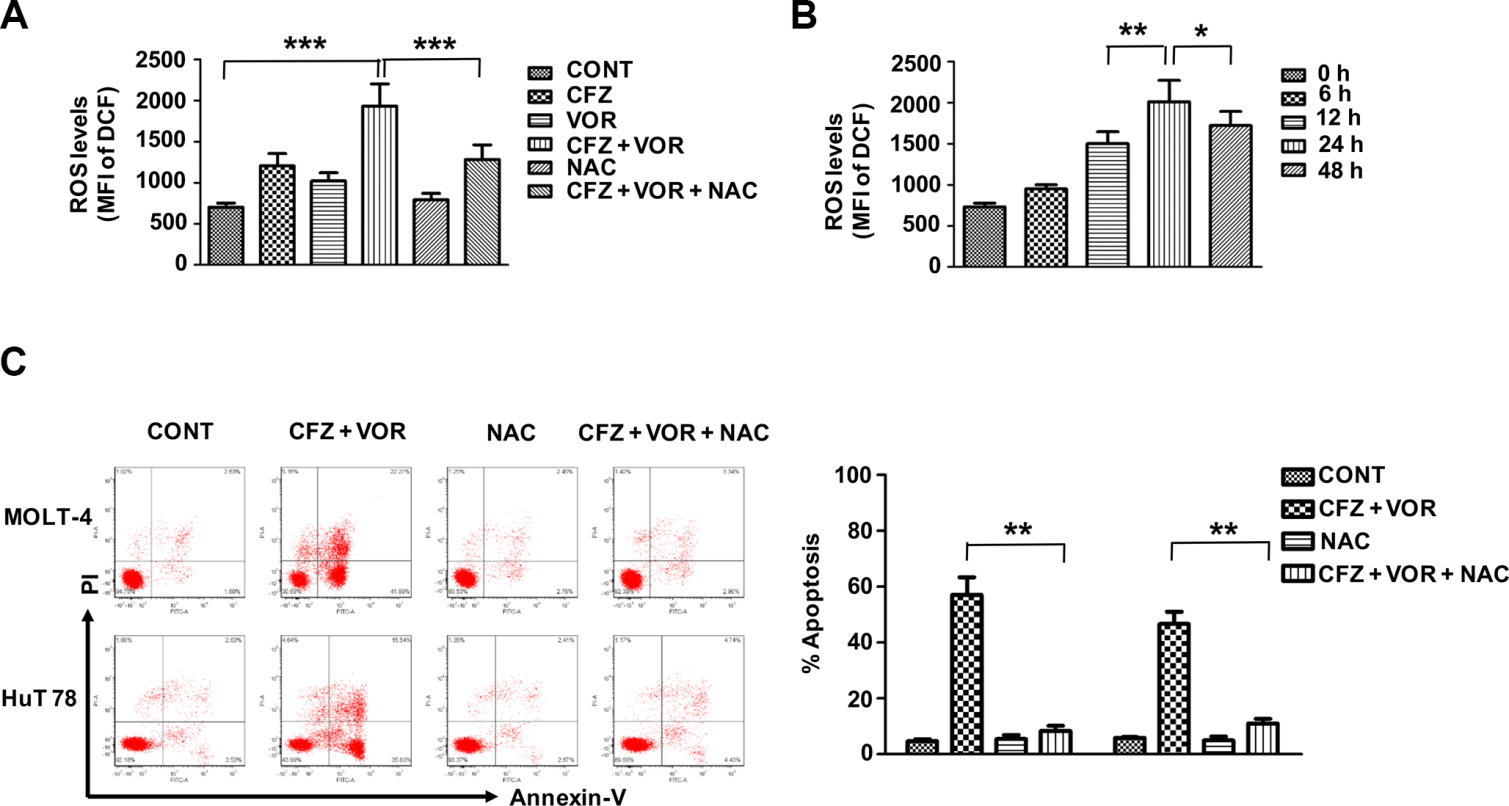
Combination treatment of carfilzomib and vorinostat induced ROS generation and induced apoptosis by the combination treatment is blocked by the ROS inhibitor (**A**) MOLT-4 cells were pre-incubated with or without 10 mM NAC for 3 h and then treated with carfilzomib (6 nM) or/and vorinostat (0.4 μM) for 24 h, then the level of ROS was detected by flow cytometry using DCFH-DA. (**B**) MOLT-4 cells were treated with carfilzomib (6 nM) and vorinostat (0.4 μM), then the level of ROS was detected at various intervals by flow cytometry using DCFH-DA. (**C**) Cells were pre-incubated with or without 10 mM NAC for 3 h and then treated with carfilzomib (MOLT-4 6 nM, HuT 78 8 nM) and vorinostat (MOLT-4 0.4 μM, HuT 78 0.4 μM) for 48 h, then cell apoptosis was monitored by Annexin V/PI staining. Columns represent the average percent of Annexin V positive cells from three independent experiments, which are shown as the mean ± SD. CFZ, carfilzomib; VOR, vorinostat. *represent *p* < 0.05; **represent *p* < 0.01; ***represent *p* < 0.001.

Increased reactive oxygen species (ROS) levels have previously been shown to play an important role in the induction of apoptosis resulting from the combinatorial treatment of a proteasome inhibitor with an HDACI [38, 39], we next evaluated ROS production in treated MOLT-4 cells by flow cytometry using DCFH- DA. Treatment with either carfilzomib or vorinostat alone slightly increased the level of ROS. However, the combination of carfilzomib with vorinostat markedly increased ROS generation (Figure 4A). Increases in ROS were observed starting from the treatment of 12 h, with maximal increase at 24 h (Figure 4B). Importantly, the ROS scavenger N-acetylcysteine (NAC) largely abrogated ROS generation (Figure 4A) and dramatically attenuated cell apoptosis induced by the combination treatment (Figure 4C). These findings indicate that the induced apoptosis by combination treatment of carfilzomib with vorinostat is mediated through the increase of ROS in T-cell leukemia/ lymphoma cells.

### p38MAPK and JNK activation contribute to carfilzomib and vorinostat-induced apoptosis

**Figure 5:**
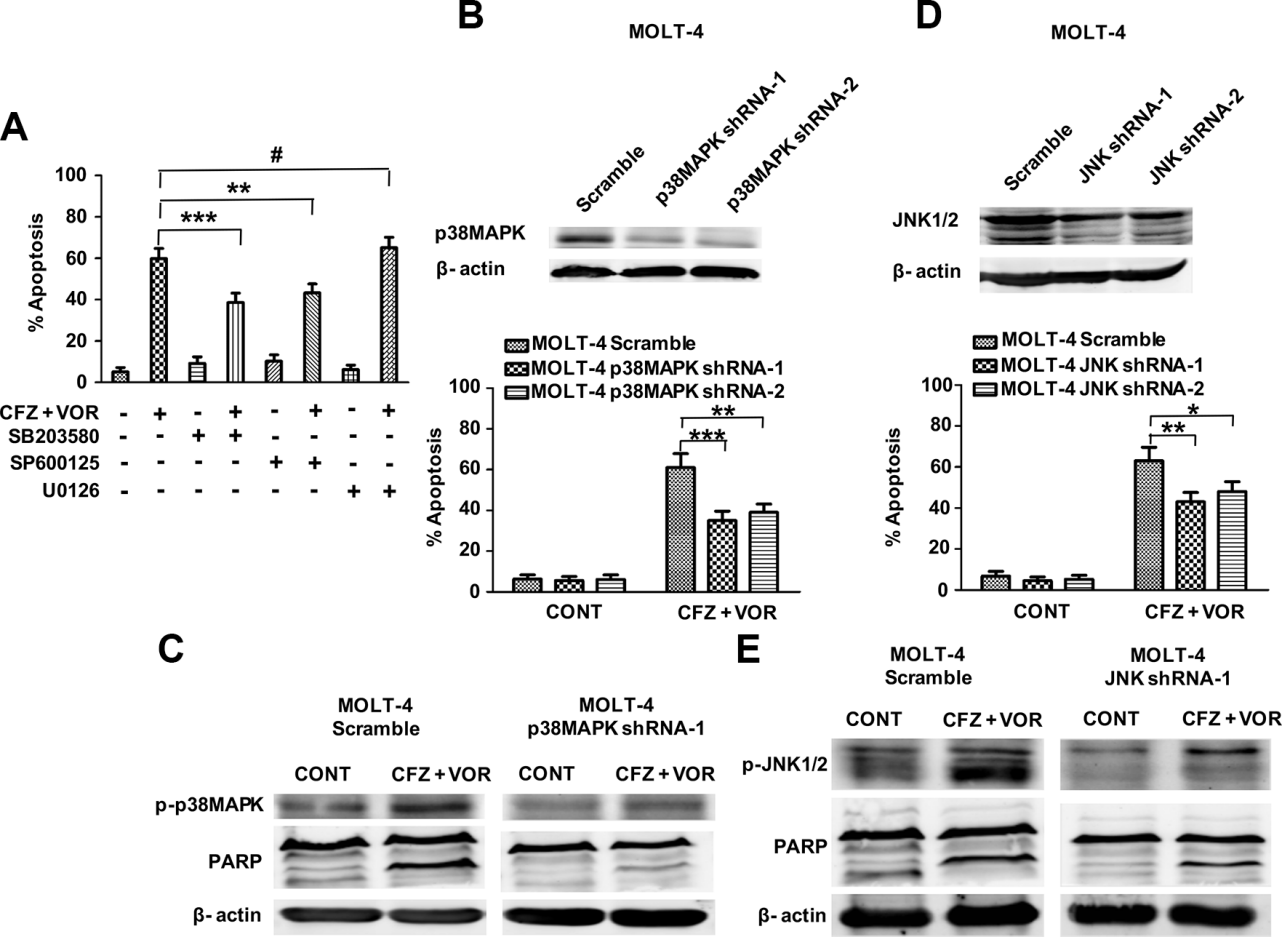
The activation of p38MAPK and JNK contribute to carfilzomib and vorinostat-induced apoptosis (**A**) Pretreatment with U0126, SB203580, and SP600125 (10 μM each) for 2 h, after which MOLT-4 cells were treated with 6 nM carfilzomib or/and 0.4 μM vorinostat for 48 h, then cell apoptosis was monitored by Annexin V/PI staining. (**B**) MOLT-4 cells stably expressing p38MAPK shRNA or scrambled sequence were treated with 6 nM carfilzomib or/and 0.4 μM vorinostat for 48 h, then cell apoptosis was monitored by Annexin V/PI staining. Inset: Relative expression of p38MAPK protein in MOLT-4 cells stably expressing p38MAPK shRNA and scrambled sequence. (**C**) After treatment as in B, the expression of p-p38MAPK and PARP proteins was monitored by western blot. (**D**) MOLT-4 cells stably expressing JNK shRNA or scrambled sequence were treated as in B, then cell apoptosis was monitored by Annexin V/PI staining. Inset: Relative expression of JNK protein in MOLT-4 cells stably expressing JNK shRNA and scrambled sequence. (**E**) MOLT-4 cells stably expressing JNK shRNA or scrambled sequence were treated as in B, and then the expression of JNK and PARP proteins was monitored by western blot. * represent *p* < 0.05; ** represent *p* < 0.01; *** represent *p* < 0.001; ^**#**^ represent *p* > 0.05.

To determine whether the activation of ERK1/2, p38MAPK, and JNK is involoved in apoptosis induced by combination treatment of carfilzomib and vorinostat, cells were pretreated with 10 μM of the inhibitors U0126 (ERK1/2), SB203580 (p38MAPK), and SP600125 (JNK) for two hours prior to combination treatment. JNK pathway inhibitor SP600125 and p38MAPK pathway inhibitor SB203580 partially protected cells from carfilzomib and vorinostat-induced apoptosis (Figure 5A). However, ERK1/2 pathway inhibitor U0126 did not attenuate cell apoptosis induced by combination treatment (Figure 5A). To further explore the impact of the p38MAPK and JNK pathways on the sensitivity of T-cell leukemia/lymphoma cells to the combination treatment, we examined the effect of the combined inhibitors in the MOLT-4 cells with different levels of p38MAPK or JNK. MOLT-4 cells were stably transduced with the lentivirus encoding either p38MAPK shRNA or JNK shRNA and displayed a clear reduction in p38MAPK or JNK expression, respectively, compared with cells transduced with scrambled sequence controls (Figure 5B and 5D). p38MAPK shRNA cells were significantly less sensitive than control cells to carfilzomib/vorinostat lethality (Figure 5B). Notably, p38MAPK shRNA cells attenuated carfilzomib/vorinostat-mediated p38MAPK activation and PARP cleavage compared with control cells (Figure 5C). Similar results were observed in JNK shRNA cells (Figure 5D and 5E). Together, these data show that activation of p38MAPK and JNK contributes to cell apoptosis induced by the combination treatment of carfilzomib and vorinostat.

### The relationship between ROS and the pathways of p38MAPK and JNK

**Figure 6:**
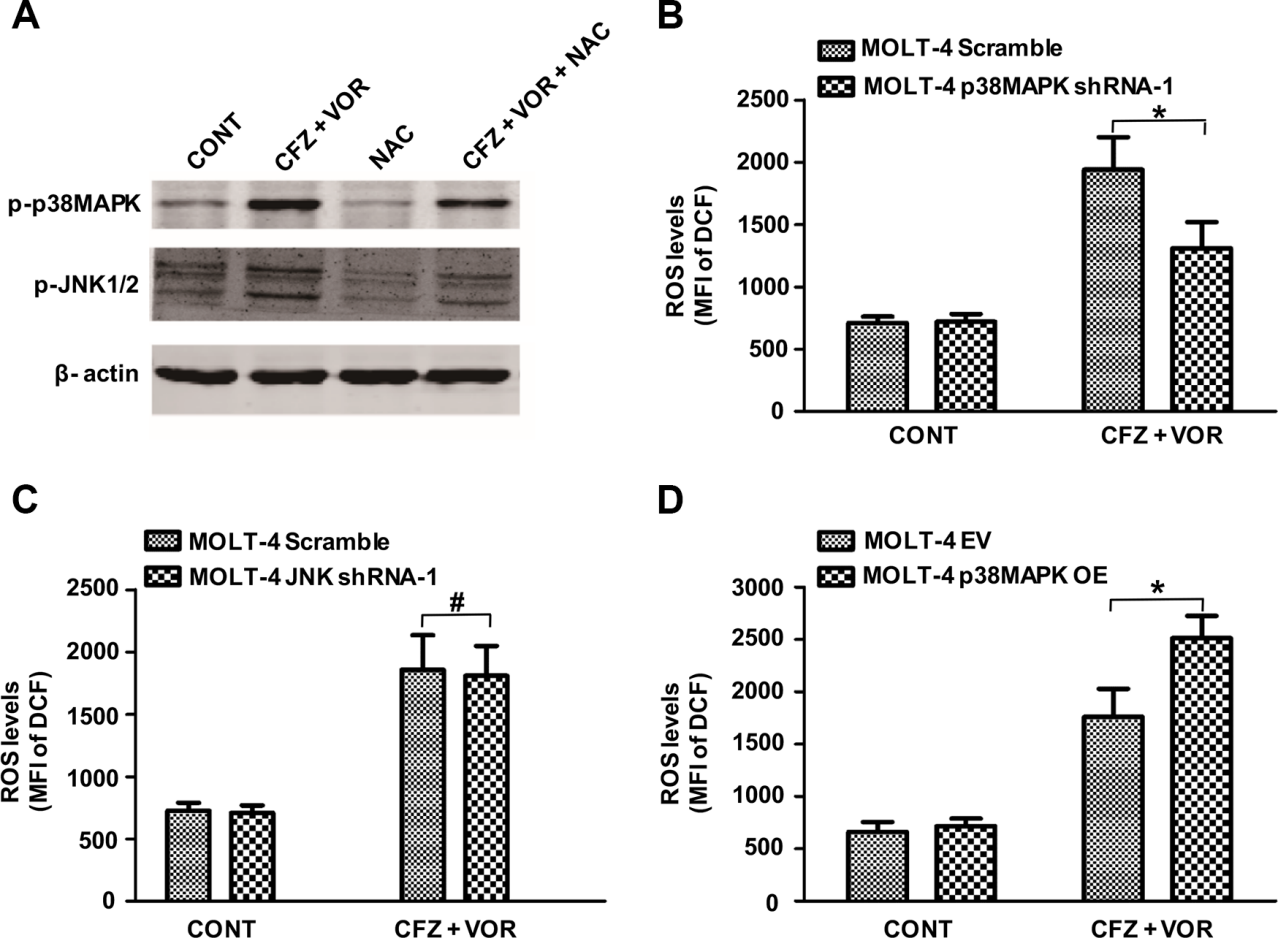
An amplification loop exists between ROS and p38MAPK pathway but not JNK pathway (**A**) MOLT-4 cells were pre-incubated with or without 10 mM NAC for 3 h and then treated with carfilzomib (6 nM) and vorinostat (0.4 μM) for 48 h, then the expression of p-p38MAPK and p-JNK proteins was monitored by western blot. (**B**) MOLT-4 cells stably expressing p38MAPK shRNA or scrambled sequence were treated with 6 nM carfilzomib and 0.4 μM vorinostat for 48 h, then the level of ROS was detected using DCFH-DA. (**C**) MOLT-4 cells stably expressing JNK shRNA or scrambled sequence were treated as in B, then the level of ROS was detected using DCFH-DA. (**D**) MOLT-4 cells transfected with plasmid DNA containing p38MAPK or empty vector were treated as in B, then the level of ROS was detected using DCFH-DA. *represent *p* < 0.05; ^#^ represent *p* > 0.05.

Previous studies have shown that oxidative stress can activate MAPK signaling pathways [40]. We next examined whether the activity of p38MAPK and JNK is regulated by ROS. The ROS inhibitor NAC decreased the phosphorylation of p38MAPK and JNK in MOLT-4 cells (Figure 6A). This result may implicate that p38MAPK and JNK act as a downstream effector of ROS. We further explored whether a feedback mechanism underlies between ROS and the cell signaling of p38MAPK and JNK. Compared to scrambled control cells, p38MAPK shRNA cells significantly diminished carfilzomib/vorinostat-mediated ROS generation (Figure 6B). However, the increase in carfilzomib/vorinostat-mediated ROS generation was similar in scrambled control and JNK shRNA cells (Figure 6C). Furthermore, overexpression of p38MAPK significantly increased carfilzomib/vorinostat-mediated ROS generation (Figure 6D), suggesting that an amplification loop exists between ROS and the p38MAPK pathway.

### The combination of carfilzomib with vorinostat inhibits tumor growth in a human xenograft model

**Figure 7:**
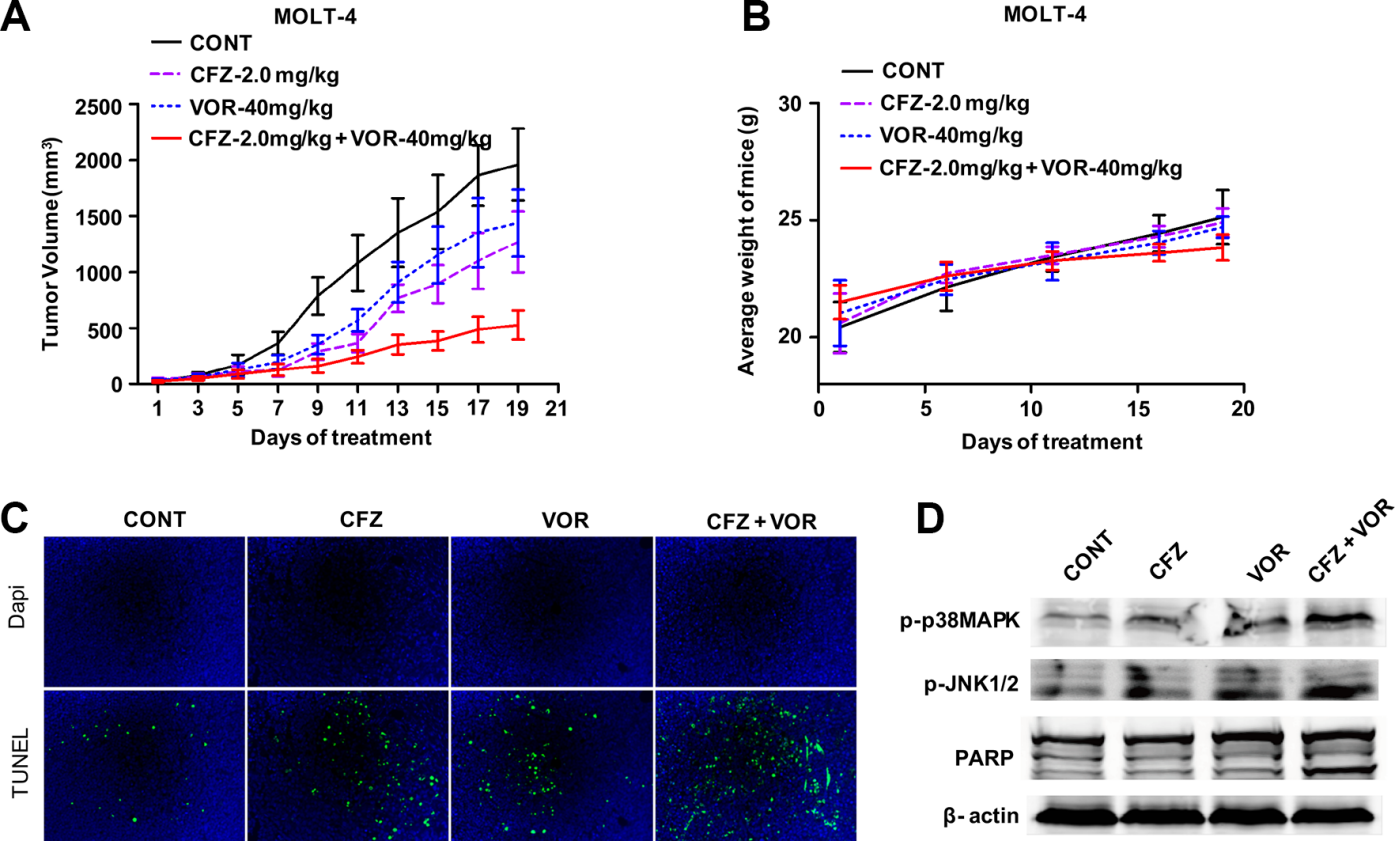
The combination of carfilzomib with vorinostat inhibits tumor growth in a human xenograft model (**A**) Nude mice (4–6 weeks old) were injected subcutaneously with 5 × 10^6^ MOLT-4 cells into the right flank. After tumors were visible, the mice received indicated doses carfilzomib with or without vorinostat as described in “Animal studies”. Tumor volume was measured every other day with calipers and calculated using the formula 0.5 × a × b^2^ in millimeters. Results represent the Mean ± SD. (**B**) The body weight of mice after treatment was measured twice every week. Results represent the Mean ± SD. (**C**) Sections from tumor samples per group were stained with FITC-dUTP as described in materials and methods (×200). (**D**) Tumor samples were homogenized and lysed. The extracted proteins were probed with western blot for p-p38MAPK, p-JNK and PARP proteins. Each lane was loaded with 30 μg of protein. The β-actin was used as an internal control.

We next assessed the functional interactions between carfilzomib and vorinostat *in vivo*. Nude mice were inoculated in the flank with 5 × 10^6^ MOLT-4 cells. After tumors were visible, mice were administered 2.0 mg/ kg carfilzomib (i.v., days 1, 2, 8, 9, 15 and 16) with or without 40 mg/kg vorinostat (i.p., day 1, 2, 3, 8, 9, 10, 15, 16 and 17). As shown in Figure 7A, carfilzomib or vorinostat alone slightly inhibited tumor growth. However combination treatment resulted in potent inhibition of tumor growth compared to single inhibitor treatment. Moreover, body weight loss was not observed in any treatment group (Figure 7B). TUNEL assay showed that combination treatment resulted in a pronounced increase of cell apoptosis compared to control and single inhibitor treatment (Figure 7C). Western blot analysis showed that combination treatment enhanced the phosphorylation of p38MAPK and JNK, and PARP cleavage compared to control and single inhibitor treatment (Figure 7D), which is consistent with *in vitro* results. Together, these results show that combination treatment with carfilzomib and vorinostat results in enhanced antitumor activity *in vivo* and is well-tolerated in animals.

### Combination treatment of carfilzomib and vorinostat induces cell apoptosis in human primary T-cell leukemia/lymphoma cells

We wish the combination treatment of carfilzomib and vorinostat will be a potential candidate treatment strategy for human primary T-cell leukemia/lymphoma. Next, we examined the effect of the combination treatment in human primary T-cell leukemia/lymphoma cells.

**Figure 8:**
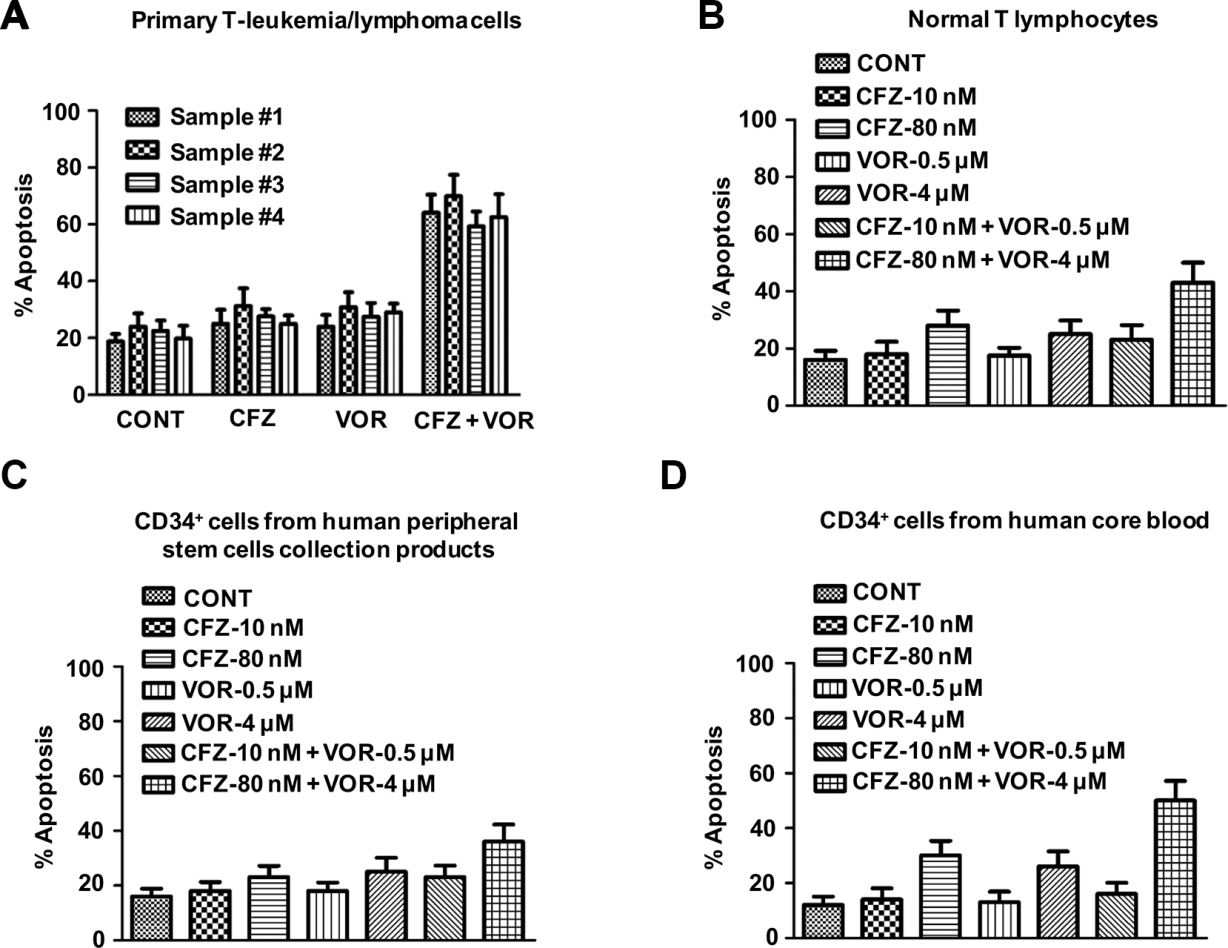
Combination treatment of carfilzomib and vorinostat induces cell apoptosis in human primary T-cell leukemia/lymphoma cells Primary T-cell leukemia/lymphoma cells (**A**), normal T lymphocytes from human peripheral blood (**B**), CD34^+^ cells from peripheral stem cells collection products (**C**) or human cord blood (**D**) were treated with indicated concentration of carfilzomib and vorinostat for 48 h, then cell apoptosis was monitored by Annexin V/PI staining.

Combination treatment with 10 nM carfilzomib and 0.5 μM vorinostat (48 h) was markedly cytotoxic to cells from three primary T-cell leukemias and one primary T-cell lymphoma (Figure 8A). Parallel studies were performed in normal T lymphocytes from human peripheral blood, CD34^+^ cells from human cord blood, or peripheral stem cell collection products in order to check the toxicity of the inhibitors. Normal hematopoietic cells were unresponsive to the combination treatment with 10 nM carfilzomib and 0.5 μM vorinostat. Moreover, individual and combination exposure to very high concentrations of carfilzomib and vorinostat (80 nM with 5 μM, respectively) resulted in relatively modest lethality toward these normal hematopoietic cells (Figure 8B–8D). These results are consistent with our xenograft data and further support that the combination treatment of carfilzomib and vorinostat potentiates their individual antitumour activity in human T-cell leukemia/lymphoma.

## DISCUSSION

The results of our present study provide evidence that carfilzomib and vorinostat cooperate to kill human T-cell leukemia/lymphoma cells *in vitro* and *in vivo*. This study was prompted by several considerations. First, synergistic interactions between bortezomib and vorinostat in several types of tumor cells, including hematopoietic cells, have been described. However, bortezomib efficacy can be limited because of peripheral neuropathy and the existence and development of resistance [28]. Second, carfilzomib is an irreversible proteasome inhibitor, which is active in bortezomib-resistant tumor cells *in vitro* and in patients with bortezomib-resistant disease. The combination of carfilzomib with vorinostat holds promise to be more efficacious and safer than the combination of bortezomib and vorinostat. Third, the combination of carfilzomib with vorinostat has been scarcely explored and currently has only been described in diffuse large-B-cell lymphoma, mantle cell lymphoma, and recently by our lab in one T cell leukemia cell line *in vitro*.

While either carfilzomib or vorinostat induce ROS generation, combination treatment enhances ROS generation considerably. Notably, cell apoptosis induced by the combination treatment was almost completely blocked by the ROS inhibitor NAC, suggesting that the induced apoptosis by the combination treatment is mediated through ROS. Previous studies have implicated increased ROS in the lethal combination of proteasome inhibition with HDACIs in many tumor cells, such as malignant human glioma cells [41], mantle cell lymphoma cells [42], cutaneous T cell lymphoma [43], leukemia cells [39] and non-small cell lung cancer cells [44]. Our findings are consistent with these and further indicate that ROS played an important functional role in the functional interaction between carfilzomib and vorinostat.

The activation of MAPK signaling pathway has been critical for a variety of toxic stimuli in the induction of apoptosis. JNK activation was previously observed in diffuse large-B-cell lymphoma (DLBCL) cells exposed to carfilzomib and HDACIs [33] and multiple myeloma cells exposed to bortezomib and NPI-0052 [45]. The activation of p38MAPK has resulted from synergy between bortezomib and valproic acid in leukemia cells [23] and between bortezomib and SAHA in cutaneous T cell lymphoma [43]. In keeping with those findings, we found that exposure of MOLT-4 and HuT 78 cells to carfilzomib and vorinostat activated p38MAPK and JNK. Further, pharmacologic or genetic JNK or p38MAPK inhibition significantly blocked carfilzomib/vorinostat-induced lethality, indicating that JNK and p38MAPK play a functional role in cell apoptosis. We also observed that ERK1/2 was activated in MOLT-4 and HuT 78 cells exposed to carfilzomib and vorinostat. Activation of ERK1/2 has been shown to be a mediator of antiapoptotic and prosurvival actions in numerous cancer model systems [5]. Previous studies reported that coadministration of the MEK inhibitor sensitizes tumor cells and human tumor xenograft models to HDACIs, suggesting that ERK1/2 inhibition is a requirement for optimal HDACI effects [46–48]. In our study, combination treatment not only failed to result in lowered levels of p-ERK1/2 in either cell line tested, but also carfilzomib alone modestly activated ERK1/2 levels and this effect was enhanced by vorinostat, indicating that inhibition of the ERK1/2 pathway is not an absolute requirement for the action of HDACIs. It has been observed that prolonged activation of Raf/MEK/MAPK pathway can exert a pro-apoptotic effect in a manner that depended upon the cellular context [49]. Dasmahapatra *et al.* [32, 33] found, however, that phosphorylation level of ERK1/2 is high in DLBCL cell lines treated by combination of carfilzomib with vorinostat, but phosphorylation level of ERK1/2 is low in Mantle cell lines treated by combination of carfilzomib with vorinostat. Although both are B lymphoma cells, the activation of ERK1/2 was different between the two cell types under identical conditions, suggesting that the involvement of ERK1/2 is even cancer subtype dependent. We further targeted ERK1/2 but failed to attenuate carfilzomib/vorinostat lethality, suggesting that although ERK1/2 pathway is a target of these drugs, it is not required to mediate their apoptosis effects.

It has been described that the activation of MAPK signaling pathway was at least in part through oxidative stress caused by increased ROS [40]. In keeping with those findings, NAC inhibits the activation of p38 and JNK. Interestingly, genetic p38MAPK inhibition diminished carfilzomib/vorinostat-mediated ROS generation. However, genetic JNK inhibition didn't exert similar effect. Furthermore, overexpression of p38MAPK significantly increased carfilzomib/vorinostat-mediated ROS generation. Thus, we propose that an amplification loop exists between ROS and p38MAPK pathway. The activation of p38MAPK pathway serves to amplify oxidative injury caused by increased ROS and, by extension, the apoptotic response.

Several lines of evidence show that combination of carfilzomib with vorinostat preferentially targets transformed cells versus normal hematopoietic cells [32, 33]. Our findings demonstrate that the combination of carfilzomib with vorinostat induced pronounced apoptosis toward both cultured and primary T-cell leukemia/lymphoma cells but exhibited minimal toxicity in normal hematopoietic cells. These findings reinforce the notion that this regimen displays minimal toxicity toward normal hematopoietic cells and selectively target transformed cells.

**Figure 9:**
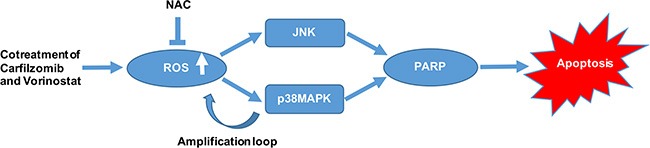
A schematic diagram showing the molecular mechanisms of apoptosis induction by carfilzomib and vorinostat in T-cell leukemia/lymphoma cells

We examined the *in vivo* effect of carfilzomib and vorinostat in a human xenograft model. Our finding suggested that 2.0 mg/kg carfilzomib by itself modestly inhibited tumor growth, whereas 40 mg/kg vorinostat by itself had little effect on tumor growth. However, combination of 2.0 mg/kg carfilzomib with 40 mg/kg vorinostat markedly inhibited tumor growth in the xenograft model. These results may have particular significance in view of the generally poor chemo-responsiveness of T-cell leukemia/lymphoma. Of note, no apparent toxicity was observed in any treatment group. Consistent with the mechanism observed *in vitro*, enhanced p38MAPK and JNK phosphorylation and increased cleavage of PARP were observed in tumor samples obtained from mice treated with carfilzomib and/or vorinostat *in vivo*. Collectively, our data show that carfilzomib and vorinostat induce apoptosis in T-cell leukemia/lymphoma cells in a highly cooperative manner, and can do so in bortezomib-resistant cells as well as primary tumor cells. We also demonstrate that ROS activates p38MAPK and JNK signaling pathways, further showing an amplification loop between ROS and p38MAPK. Activation of both of these pathways plays a significant role in the functional interaction between carfilzomib and vorinostat (Figure 9). Finally, the ability of carfilzomib/vorinostat to inhibit T-cell leukemia/lymphoma cells growth in an *in vivo* xenograft model supports further consideration of second-generation proteasome inhibitors and HDACIs use in T-cell leukemia/lymphoma cancers.

## MATERIALS AND METHODS

### Cells

T-cell leukemia (MOLT-4) and lymphoma (HuT 78) cell lines were purchased from the Cell Resource Center of Shanghai Institute for Biological Sciences (Shanghai, China). Bortezomib-resistant MOLT-4 and HuT 78 were obtained by selecting sensitive cells in the presence of nanomolar levels of bortezomib. All cell lines were cultured in RPMI-1640 media (Invitrogen, Frederick, MD), supplemented with 10% fetal bovine serum (FBS), 1% penicillin (100 units/ml), and 1% streptomycin (100 μg/ ml). Cells were maintained at 37°C in an atmosphere of 5% CO_2_ and 95% air. After informed consent in accordance with the Helsinki Declaration, patient leukemia cells were isolated from the bone marrow of three T-cell leukemia patients and lymphoma cells were obtained from the lymphoma node of one T-cell lymphoma patients. Normal T-lymphocytes were isolated from human peripheral blood using a Pan T Cell Isolation Kit II (Miltenyi Biotec Inc., CA, USA). CD34^+^ cells were isolated from human peripheral stem cell collection products or human cord blood using CD34 Progenitor Cell Isolation Kit (Miltenyi Biotec Inc., CA, USA). These studies have been approved by the institutional review board of Shanghai Tenth People's Hospital.

### Reagents

Carfilzomib was purchased from Onyx Pharmaceuticals (South San Francisco, CA, USA). Vorinostat was from Merck & Co., Inc (Rahway, NJ, USA). Bortezomib was from Millennium Pharmaceuticals (Cambridge, MA). N-acetyl-L-cysteine (NAC) was from Sigma-Aldrich (St Louis, MO). The JNK inhibitor SP600125 and p38MAPK inhibitor SB203580 were from Selleckchem (Houston, Tx). The ERK1/2 inhibitor U0126 was from Cell Signaling Technology (Beverly, MA). All other agents except NAC were formulated in dimethyl sulfoxide. NAC was dissolved in double-distilled H_2_O (ddH_2_O). The mitochondrial membrane potential assay kit with JC-1 and reactive oxygen species assay kit were from Beyotime Institute of Biotechnology (Haimen, China). The Cell Counting Kit-8 (CCK-8) was from Dojindo (Mashikimachi, Japan). The Annexin V/propidium iodide (PI) staining kit was from BD Pharmingen (Franklin Lakes, NJ).

### Cell viability and apoptosis assay

Cell viability was monitored by CCK-8. Alternatively, cell apoptosis was assessed by a flow cytometer with Annexin V/PI staining kit. Annexin V positive cells were considered apoptotic cells. Results of CCK-8 and Annexin V/PI assays were consistent.

### Cell cycle analysis

Cells were suspended with ice-cold PBS, fixed in 70% ethanol at −20°C for 18 h, after which, cells were washed with PBS and stained for 15 min at 37°C with 500 μL of 50 μg/mL propidium iodide (containing 50 μg/mL RNase) (BD Pharmingen) followed by flow cytometric analysis.

### Western blot analysis

Cells were seeded in a 6-well plate at a density of 2 × 10^5^/ml. Cell lysates were prepared from whole cell pellets. Equal amounts of proteins (30 μg per lane) were separated on 10% or 15% SDS-PAGE, transferred to nitrocellulose membrane, blocked for 1 h with 5% milk or 5% BSA, and probed with primary antibodies overnight at 4°C. Primary antibodies against various proteins were as follows: anti-phospho (p)-AKT, anti-AKT, anti-(p)-JNK, anti-JNK, anti-(p)-ERK1/2, anti-ERK1/2, anti-(p)-p38MAPK, anti-p38MAPK, anti-caspase-8, anti-caspase-9, anti-caspase-3, anti-PARP, and anti-β-actin antibodies. All antibodies were from Cell Signaling Technology (Beverly, MA). Membranes were washed three times for 10 min each with Tween 20-PBS and incubated for 1 h with Fluorescence-conjugated goat anti-mouse or anti-rabbit IgG secondary antibodies. Membranes were washed with Tween 20-PBS three times for 10 min each and developed using the Odyssey two-color infrared laser imaging system (LI-COR Bioscience, Lincoln, NE). The signal generated by β-actin was used as an internal control.

### Gene silencing

p38MAPK and JNK targeting oligonucleotide sequences were as follows: p38MAPK shRNA-1, 5′- CAAG GTCTCTGGAGGAATTCA -3′; p38MAPK shRNA-2, 5′-GCACCATGAAGATCAAGATTT -3′; JNK shRNA-1, 5′- GAGTCGGTTAGTCATTGATAG -3′; JNK shRNA-2, 5′- GTGTCTTCAATGTCAACAGAT -3′; and the shRNA control sequence, 5′- CCTAAGGTTAAGTCGCCCTCG -3′. These sequences were chemically synthesized, used for the cloning of shRNA-encoding sequences, and inserted into a lentiviral vector pLKO.1. 48 h after co-transfection with psPAX2 packaging plasmid and pMD2.G envelope plasmid into HEK-293T cells using Lipofectamine 2000 (Invitrogen, USA), the media containing lentiviral particles was harvested and used for infection of MOLT-4 cells in presence of 8 μg/ ml polybrene (Sigma-Aldrich, St. Louis, MO, USA).

### p38MAPK overexpression by plasmid transfection

The cDNA sequence of p38MAPK was obtained from GenBank (NM_001315.2). It was subcloned into pcDNA3.1 and confirmed by sequencing. An empty construct pcDNA3.1 was used as a control. Cell transfections were performed using Lipofectamine 2000 reagent (Invitrogen) following the manufacturer's instruction.

### Assessment of ROS generation

Cells were pretreated with or without NAC at 37°C for 15 min, then treated with various drugs for the indicated intervals to detect changes in the levels of ROS. Cells were washed with PBS and incubated with 10 μM 2′, 7′-dichlorodihydrofluorescein diacetate (DCFH-DA) at 37°C for 20 min and fluorescent intensity was assessed using a flow cytometer (BD, Franklin Lakes, NJ).

### Analysis of mitochondrial membrane potential

The loss of mitochondrial membrane potential was monitored by flow cytometry using mitochondrial membrane potential assay kit with JC-1. Cells were washed with PBS and stained with JC-1 dye according to the manufacturer's instructions.

### Terminal deoxynucleotidyl transferase-mediated dUTP nick end labeling (TUNEL) assay

Cell apoptosis *in vivo* was examined by a terminal deoxynucleotidyl transferase-mediated dUTP nick end labeling (TUNEL) assay as the manufacturer's protocol (Promega, USA). Tumor samples per group were analyzed after the 21 days of treatment.

### Animal studies

Nude mice (4–6 weeks old) were obtained from the Shanghai Laboratory Animal Center (Shanghai, China) and injected subcutaneously with 5 × 10^6^ MOLT-4 cells into the right flank (day 0). After tumors were visible, the mice received intravenous carfilzomib (2.0 mg/kg) on days 1, 2, 8, 9, 15 and 16, intraperitoneal vorinostat (40 mg/kg) on day 1, 2, 3, 8, 9, 10, 15, 16 and 17 or the combination treatment, respectively (5 mice per group). Tumor volumes were monitored every other day with calipers and calculated using the formula 0.5 × a × b^2^ in millimeters, where ‘a’ is the long diameter of the tumor and ‘b’ is the short diameter of the tumor. Mice body weights were measured periodically as an indicator of toxicity. At the twenty-first day, all mice were sacrificed individually by cervical dislocation. All animal studies have been approved by the institutional review board of Shanghai Tenth People's Hospital.

### Statistical analysis

Data analysis was conducted with SPSS 20.0 software. All data were represented as mean ± standard deviation (SD). Comparisons among groups were performed using one-way analysis of variance (ANOVA) with the Student-Newman-Keuls post hoc test. A *p*-value less than 0.05 was considered to be statistically significant. All experiments were performed in three or more separate experiments.
